# Multi-lab intrinsic solubility measurement reproducibility in CheqSol and shake-flask methods

**DOI:** 10.5599/admet.698

**Published:** 2019-06-05

**Authors:** Alex Avdeef

**Affiliations:** in-ADME Research, 1732 First Avenue, #102, New York, NY 10128, USA

**Keywords:** Interlaboratory solubility measurement errors, aqueous intrinsic solubility, shake-flask solubility, potentio- metric solubility, thermodynamic solubility, Henderson-Hasselbalch equation

## Abstract

This commentary compares 233 CheqSol intrinsic solubility values (log S_0_) reported in the Wiki-pS_0_ database for 145 different druglike molecules to the 838 log S_0_ values determined mostly by the saturation shake-flask (SSF) method for 124 of the molecules from the CheqSol set. The range of log S_0_ spans from -1.0 to -10.6 (log molar units), averaging at -3.8. The correlation plot between the two methods indicates r^2^ = 0.96, RMSE = 0.34 log unit, and a slight bias of -0.07 log unit. The average interlaboratory standard deviation (SD_i_) is slightly better for the CheqSol set than that of the SSF set: SD_i_^CS^ = 0.15 and SD_i_^SSF^ = 0.24. The intralaboratory errors reported in the CheqSol method (0.05 log) need to be multiplied by a factor of 3 to match the expected interlaboratory errors for the method. The scale factor, in part, relates to the hidden systematic errors in the single-lab values. It is expected that improved standardizations in the ‘gold standard’ SSF method, as suggested in the recent ‘white paper’ on solubility measurement methodology, should make the SD_i_ of both methods be about ~0.15 log unit. The multi-lab averaged log S_0_ (and the corresponding SD_i_) values could be helpful additions to existing training-set molecules used to predict the intrinsic solubility of drugs and druglike molecules.

## Introduction

This commentary considers the *inter*laboratory reproducibility of published aqueous intrinsic solubility data (log *S*_0_) for 124 drugs, determined both by the potentiometric CheqSol (CS) method (233 reported log *S*_0_^CS^ values) and mostly by the ‘gold standard’ saturation shake-flask (SSF) method (838 log *S*_0_^SSF^). For each drug, its method-dependent *inter*laboratory measurement standard deviations (*SD*_i_^CS^ and *SD*_i_^SSF^) are estimated, by comparing solubility values for a given drug determined in different laboratories. The multi-lab averaged log *S_0_* (and the corresponding *SD_i_* values) could be helpful additions to existing training-set molecules used to predict the intrinsic solubility of drugs and druglike molecules.

The present contribution is the third in a series of papers aiming to address contemporary issues of solubility measurement, interpretation and prediction [1,2]. These are intended to serve as prologue/accompaniment to an upcoming session on solubility at the IAPC-8 meeting in Split, Croatia, 9-11 September 2019. Since 2009, the International Association of Physical Chemists (IAPC, www.iapchem.org) series of symposia maintained extensive coverage of the topic of solubility measurement, both from solid state and solution perspectives. At the 2015 IAPC-4 meeting, the special session on solubility measurement resulted in a widely-circulated ‘white paper’ drawing on expert consensus thoughts of scientists from six countries (Hungary, Russia, Serbia, Spain, Sweden, United States) [3]. It is expected that future sessions will continue to cover solubility methods and strategies, to critically address the different needs in pharmaceutical research, spanning from drug discovery to drug development.

## Background

Most of the small-molecule research compounds in today’s drug discovery projects are ionizable and poorly soluble in water, and are thus prone to show low and/or erratic *in vivo* intestinal absorption [4]. In discovery, high-throughput microtitre plate methods are used to estimate solubility, where small volumes of 10 mM DMSO solutions of library compounds are added to buffer solutions to induce formation of solid suspensions in the wells. Such estimates of solubility are needed, in part, to anticipate whether compounds would precipitate in bioassays (and thus indicate false positives). In parallel, methods to predict solubility of molecules play an important role in drug discovery, since virtual screening of compound libraries could prioritize molecules for testing in early *in vitro* screens [2,5,6].

### Saturation shake-flask (SSF) and potentiometric (CheqSol) methods

In more advanced stages of drug research, solubility measurement necessarily becomes more rigorous, where ‘solubility’ refers to the concentration of a solute in a saturated solution, where the dissolved molecule is in a thermodynamic equilibrium with its crystalline form suspended in the solution (of a known pH, composition, and temperature). Accurate solubility measurement of druglike molecules can be very difficult to do well, although to an untrained eye it ought to be as easy as measuring the concentration of a molecule in water. In development projects, thermodynamic solubility measurements are usually done using some variant of the SSF method [3]. As an alternative, a potentiometric procedure, called the dissolution template titration (DTT) method was introduced in 1998 [7] and validated two years later [8]. The methodology is capable of producing highly precise (*intra*laboratory SD ~0.07 log unit) measurements of aqueous intrinsic solubility, log *S*_0_, *i.e*., the solubility of the uncharged form of an ionizable molecule. A much faster variant of the pH-titration method, called CheqSol, was described in 2005 [9]. Instruments implementing the potentiometric methods have been used in several universities and pharmaceutical companies.

All of the potentiometric methods require that the molecule be ionizable and that the accurately-measured p*K*_a_ be provided. The molecule cannot be *too* soluble, since the method depends on the pH difference between a saturated and an unsaturated solution in the titration where the molecule is half ionized. So, it is ideally suited for low-soluble molecules, since these molecules display large pH differences. Furthermore, to calculate the CheqSol log *S*_0_, it is assumed that solubility as a function of pH follows the curve predicted by the Henderson-Hasselbalch equation. The molecule needs to be stable to hydrolysis when repeatedly exposed to pH conditions far from neutral. Means to recognize hydrolytic decomposition are important to incorporate into the measurement. Sometimes multiple polymorphs may form in the CheqSol method, which requires solid state characterization to identify. In the traditional SSF method, most of the time, the thermodynamically most-stable form of the solid is the one associated with the measured solubility [3]. Equilibration times are selected to be long (24-168 h) to ensure this expectation.

### Challenging measurement

What can make solubility measurement of an ionizable low-soluble molecule so difficult? There are two sides to consider for the reaction at equilibrium: (a) the solid state and (b) the solution. Temperature needs to be regulated and specified. To keep the following examples simple, let’s assume that the solvent is distilled water or an aqueous buffer, and that the crystalline form of the test compound is a free-acid/base or a salt. Multiple circumstances may arise, some making the interpretation of the measurement challenging: (i) the simplest suspension is the one where no drug ionization takes place on equilibration. One needs to measure the concentration of the compound in the saturated solution, and to confirm that the solid state form is unchanged – simple. (ii) However, if the solid introduced is not the thermodynamically most-stable polymorph (or is amorphous), then it is possible that the measured solubility would correspond to a different solid form. That’s important to know. (iii) Complication can arise if a low-soluble weak base is added to water (usually saturated with CO_2_ from the air). The pH will change, depending on the p*K*_a_ of the molecule. The ambient CO_2_ may act as a buffer, so the final pH of the saturated solution needs to be carefully measured. Otherwise, the calculated log *S*_0_ could be quite erroneous. (iv) If a solid salt of the compound is added to water, a supersaturated solution may form. On equilibration, there could be two precipitates in the suspension: the original compound salt and the neutral free-acid/base form of the solid (at the pH called ‘pH_max_’). Solid state characterization of the solid(s) and the measurement of pH would be highly beneficial. (v) When a buffer medium is used, the analysis of the solution and solid states can be complicated, as water-soluble drug-buffer complexes or aggregates may form [10]. In a supersaturated solution, drug molecules may self-associate as sub-micellar aggregates, particularly if they are surface-active [11,12]. For bases introduced as drug salts into a high-pH solution, the charged drug in the supersaturated solution can disproportionate into oil or undergo precipitation into an amorphous solid, along with which charged water-soluble aggregates may co-exist [10-13]. Given enough time, the multiple phases are expected to undergo transformation into a thermodynamically most-stable crystalline solid. Good understanding of solution chemistry and solid state characterization is essential for correctly interpreting the results of solubility measurements, so that high-quality intrinsic solubility data can be reported [3].

### The need for high-quality data in accurate solubility prediction

Accurate prediction of the intrinsic solubility of *druglike* molecules [2] requires that (a) the log *S*_0_ values used to train the prediction method are of high quality (with water solubility values, log *S*_w_, or values measured at a particular pH, log *S*_pH_, properly corrected for ionization [14] and with all solubility values referring to the same temperature [15]), and (b) the compounds in the training set cover the *druglike* chemical space of the test set of compounds.

These two important notions became the focus of a number of studies since 2008, spurred by the publications of Llinàs *et al*. [16] and Hopfinger *et al*. [17]. These authors introduced the ‘Solubility Challenge,’ a competition to probe the limits of prediction methods. The CheqSol method was used to measure the log *S*_0_ of 132 structurally diverse drugs. The log *S*_0_ values of 100 molecules were offered as the training set for the prediction of an external test set of 32 molecules (not found in the training set), whose values were not revealed before the completion of the competition.

In a number of earlier studies, it was suggested that the typical error in measured aqueous solubility is ~0.6 log unit or higher, when the solubility values were collected from many published sources [18]. This suggested that the quality of prediction methods was approaching the experimental limit. However, in the Solubility Challenge competition all of the values came from one laboratory, and the *intra*laboratory precision (repetitive measurements of the same sample by the same chemist, using the same instrument) of the CheqSol data was reported to be SD = 0.05 log unit. It was not known what the expected *inter*laboratory precision would be, given the unknown systematic errors that might affect the accuracy of results. For example, when 125 published CheqSol values were compared in 2015 to those obtained by the SSF method, it was reported that *r^2^* = 0.90, prediction root-mean-square-error, *RMSE* = 0.52 log unit, and there was a slight bias of -0.13 log unit [19]. The values in the comparison came from the Wiki*-*p*S*_0_ database (*in-ADME* Research), which at that time contained 4557 log *S*_0_ entries. The database now has 6355 entries, with many newly added CheqSol and SSF values. It was thus of interest to update and better characterize the comparison of data quality between the CheqSol and the ‘gold standard’ saturation shake-flask methods. In parallel, armed with new curated data, the *second* Solubility Challenge has just been announced [2], with the prediction submission deadline set to 8 September 2019, the day before the IAPC-8 conference starts. The Excel submission form in *Supporting Info* at https://pubs.acs.org/doi/suppl/10.1021/acs.jcim.9b00345 is freely downloadable for those interested to participate. The data described below could be a useful addition to other druglike training sets currently in circulation.

## Method

### Data source: Wiki-pS_0_ database

The ongoing *Wiki-pS_0_* database project [2,3,15,19], which started in 2011, now has 6355 log *S*_0_ entries for 3014 different drug-relevant molecules (solids at room temperature), drawing on the study of 1325 publications. The overall *inter*laboratory standard deviation, *SD*_i_^ALL^ = 0.17 log unit, has been estimated from the 870 molecules for which solubility was reported from two or more different sources (comprising 4209 individual *S*_0_ values), by taking the average of the individual 870 SD values. The *SD*_i_^ALL^, being lower than the older estimate of solubility measurement error (~0.6 log unit [18]), indicates that (i) when legacy data are subjected to critical analysis, as recommended in [3,15,19], improvements in the quality of the extracted log *S*_0_ data can be achieved, and (ii) there is room for further improvement to the current prediction methods. Alongside the database, the *p*DISOL-X program (*in-ADME* Research) was designed to interpret solubility data and make temperature corrections, to produce a reliable estimate of the underpinning log *S*_0_ [10,11,20].

## Results

There are 233 reported CheqSol log *S*_0_ values in the Wiki*-*p*S*_0_ database for 145 different druglike molecules. For 124 of the molecules, there are 838 reported log *S*_0_ determined mostly by the SSF method. Of the 838 entries, 298 (36 %) were log *S*_0_ values calculated from log *S* vs. pH data (using *p*DISOL-X), based on a total of 2925 individual log *S*_pH_ measurments. (For 21 of the 145 molecules, SSF data have not been located in the literature.) Table 1 lists the solubility values for the 124 overlapping molecules measured by the CheqSol and SSF methods. The range of log S_0_ spans from -1.0 to -10.6 (log molar units), averaging at -3.8.

Note that indomethacin is not listed in the table. The CheqSol value was not accepted in the Wiki-p*S*_0_ database due to the hydrolytic decomposition encountered during the CheqSol assay [30]. On the other hand, the pH-metric DDT method indicated log *S*_0_^DTT^ = -5.33 [31], in good agreement with the average of 20 *inter*laboratory SSF measurements: log *S*_0_^SSF^ = -5.49, *SD*_i_ = 0.23.

**Figure 1.**
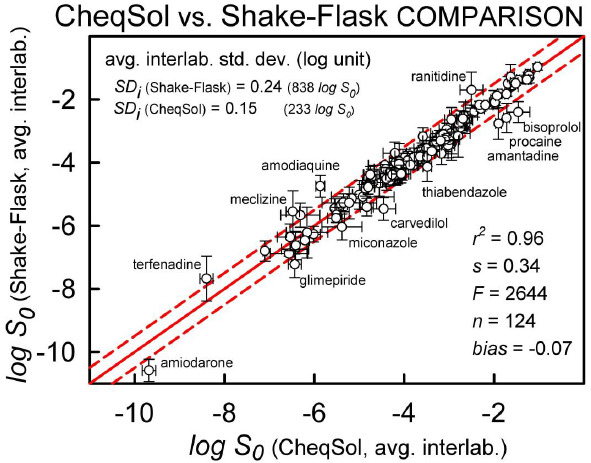
The correlation plot between published CheqSol and saturation shake-flask (SSF) intrinsic solubility values (log molar) at 25 °C. The solid diagonal line is the identity line. The dashed lines are displacements by ±0.5 log.

Figure 1 shows the correlation plot between the two types of measurements, with each point showing both method *inter*laboratory error bars. The statistics have improved slightly over the 2015 comparison [19], with current values being *r*^*2*^ = 0.96, *RMSE* = 0.34 log unit, with a lower slight *bias* of -0.07 log unit. The average *inter*laboratory standard deviation is slightly lower for the CheqSol set over that of the SSF set: *SD*_i_^CS^ = 0.15 and *SD*_i_^SSF^ = 0.24, which probably highlights the benefit of using a highly standardized method (CheqSol) over an ‘open’ method (SSF). It should be kept in mind that the above comparison sets are small. For 870 of these sorts of comparisons, *SD*_i_^ALL^ = 0.17. The *intra*laboratory comparison between the methods by one group of researchers performing both the SSF and CheqSol measurements (*both* highly-standardized) [32] produced the statistics *r*^2^ = 0.96, *RMSE* = 0.20 for 15 compounds, comparable to *SD*_i_^AL*L*^. The latter is the target for computational methods to aim at, provided that the training sets are of high and consistent quality.

## Conclusions

This brief commentary reasserts that the quality of the standardized CheqSol measurements is comparable to that of the ‘gold standard’ saturation shake-flask measurements. Measurement errors are much lower than commonly acknowledged in the computational prediction community. The *intra*laboratory (single instrument) errors reported in the CheqSol method (0.05 log) need to be multiplied by a factor of 3 to match the expected *inter*laboratory errors for the method (0.15 log). The scale factor, in part, relates to the hidden systematic errors in the single-lab values. It is expected that better standardizations in the ‘open’ SSF methods, as recommended in the ‘white paper’ [3], may equalize the *SD*_i_ of both methods at about ~0.15 log unit. When solubility prediction methods indicate *RMSE below* 0.15, ‘overfitting’ is probably taking place, overlapping noise with information.

## Figures and Tables

**Table 1.** Averaged Saturation Shake-Flask (SSF) and CheqSol (CS) Intrinsic Solubility (log molar) ^a^

Compound	avg. 25 °C log *S*_0_^SSF^	*SD^SSF^*	*N*	avg. 25 °C log *S*_0_^CS^	*SD* ^CS^	*N*	Ref. (CheqSol)
**Acebutolol**	-2.50	0.42	2	-2.68	0.31	1	[17]
**Acetaminophen**	-0.97	0.10	18	-1.03	0.05	2	[16,22]
**Acetazolamide**	-2.38	0.18	10	-2.44	0.04	1	[16]
**Alprenolol**	-2.83	0.16	1	-2.66	0.04	2	[22,28]
**Amantadine**	-2.76	0.50	1	-1.90	0.07	2	[22,29]
**Amiodarone**	-10.58	0.35	4	-9.68	0.15	1	[22]
**Amitriptyline**	-4.66	0.40	5	-4.55	0.15	1	[22]
**Amodiaquine**	-4.74	0.35	1	-5.87	0.10	2	[16,22]
**Amoxicillin**	-2.13	0.06	10	-1.97	0.08	1	[17]
**Aripiprazole**	-6.74	0.16	2	-6.43	0.30	1	[28]
**Atenolol**	-1.20	0.06	7	-1.25	0.06	4	[22,23,27,28]
**Atropine**	-2.01	0.29	4	-2.00	0.07	1	[16]
**Barbital,Hexo-**	-2.79	0.15	5	-2.67	0.07	1	[16]
**Barbital,Pheno-**	-2.30	0.08	25	-2.29	0.07	1	[22]
**Bendroflumethiazide**	-4.35	0.34	4	-4.19	0.15	2	[24,29]
**Benzocaine**	-2.18	0.12	13	-2.33	0.31	1	[17]
**Benzoic_Acid**	-1.59	0.05	12	-1.61	0.15	1	[22]
**Benzoic_Acid,4-Hydroxy-**	-1.38	0.10	5	-1.46	0.04	1	[22]
**Benzthiazide**	-4.84	0.28	4	-4.86	0.04	2	[22,24]
**Bifonazole**	-6.27	0.25	4	-6.01	0.15	1	[25]
**Bisoprolol**	-2.40	0.34	2	-1.46	0.26	1	[28]
**Bupivacaine**	-3.47	0.18	9	-3.08	0.21	2	[16,24]
**Carprofen**	-4.63	0.05	1	-4.70	0.22	1	[22]
**Carvedilol**	-5.46	0.36	12	-4.46	0.27	1	[28]
**Cephalothin**	-3.40	0.32	1	-2.94	0.04	1	[16]
**Chlorpheniramine**	-2.65	0.10	1	-2.60	0.12	3	[21,22]
**Chlorpromazine**	-5.45	0.31	11	-5.55	0.04	1	[22]
**Chlorpropamide**	-3.15	0.17	5	-3.21	0.05	2	[16,28]
**Chlorprothixene**	-5.66	0.38	3	-6.31	0.44	3	[16,22]
**Chlorzoxazone**	-2.78	0.11	3	-2.64	0.04	2	[16,29]
**Cimetidine**	-1.50	0.22	7	-1.69	0.04	1	[16]
**Ciprofloxacin**	-3.57	0.19	19	-3.60	0.18	1	[22]
**Cyproheptadine**	-5.02	0.46	4	-5.00	0.15	1	[24]
**Diazoxide**	-3.46	0.26	3	-3.36	0.07	1	[16]
**Dibucaine**	-3.70	0.35	1	-4.20	0.26	2	[17,24]
**Diclofenac**	-5.33	0.19	27	-5.40	0.14	6	[9,21,22,24,26]
**Diethylstilbestrol**	-4.38	0.38	6	-4.43	0.48	1	[17]
**Difloxacin**	-3.94	0.10	2	-3.60	0.04	1	[16]
**Diflunisal**	-5.08	0.40	5	-4.92	0.70	6	[17,21]
**Diltiazem**	-2.95	0.20	1	-3.05	0.16	2	[22]
**Diphenhydramine**	-3.29	0.64	3	-2.95	0.04	1	[22]
**Dipyridamole**	-5.13	0.13	10	-5.16	0.02	1	[17]
**Enrofloxacin**	-3.16	0.13	7	-3.18	0.18	1	[16]
**Famotidine**	-2.67	0.27	5	-2.65	0.04	1	[22]
**Fenoprofen**	-3.92	0.20	1	-3.70	0.26	1	[16]
**Flufenamic_Acid**	-5.27	0.28	10	-5.35	0.04	1	[22]
**Flumequine**	-4.10	0.08	1	-3.80	0.11	2	[16,22]
**Flurbiprofen**	-4.36	0.19	21	-4.05	0.15	2	[16,22]
**Fluvastatin**	-3.78	0.06	1	-3.87	0.16	1	[28]
**Furosemide**	-4.51	0.20	19	-4.18	0.12	3	[21,22]
**Glibenclamide**	-6.63	0.45	10	-6.41	0.07	2	[27,28]
**Gliclazide**	-4.27	0.40	14	-4.20	0.15	3	[23,27,28]
**Glimepiride**	-7.22	0.42	5	-6.44	0.15	1	[27]
**Glipizide**	-5.66	0.24	6	-5.51	0.10	3	[27-29]
**Guanine**	-4.09	0.07	2	-4.43	0.37	1	[16]
**Haloperidol**	-5.76	0.14	8	-5.52	0.19	2	[22,24]
**Hydroflumethiazide**	-2.72	0.11	15	-2.70	0.03	3	[21,29]
**Hydrochlorothiazide**	-3.08	0.08	5	-2.97	0.44	1	[16]
**Ibuprofen**	-3.80	0.22	19	-3.97	0.23	4	[21-23]
**Imipramine**	-4.39	0.28	7	-4.15	0.13	4	[17,21,22]
**Ketoprofen**	-3.45	0.23	20	-3.23	0.02	4	[17,21,24]
**Lidocaine**	-1.82	0.08	19	-1.87	0.05	1	[22]
**Loperamide**	-6.80	0.32	3	-7.10	0.04	2	[16,22]
**Maprotiline**	-4.54	0.26	3	-4.75	0.08	2	[22,24]
**Meclizine**	-5.55	0.66	1	-6.48	0.15	1	[29]
**Meclofenamic_Acid**	-6.88	0.08	2	-6.56	0.42	2	[17,22]
**Mefenamic_Acid**	-6.36	0.42	6	-6.54	0.28	2	[16,22]
**Metoclopramide**	-3.18	0.28	1	-3.58	0.07	1	[29]
**Metoprolol**	-1.31	0.16	2	-1.22	0.01	2	[22,28]
**Metronidazole**	-1.25	0.06	17	-1.22	0.04	1	[16]
**Miconazole**	-6.04	0.41	4	-5.38	0.45	2	[16,22]
**Nadolol**	-1.29	0.40	2	-1.63	0.08	2	[22,28]
**Nalidixic_Acid**	-3.76	0.26	8	-3.61	0.04	1	[16]
**Naphthoic_Acid,2-**	-3.82	0.28	5	-3.77	0.15	1	[22]
**Naphthol,1-**	-2.09	0.10	6	-1.98	0.07	1	[22]
**Naproxen**	-4.21	0.14	15	-4.41	0.25	2	[16,22]
**Niflumic_Acid**	-4.18	0.13	6	-4.11	0.08	2	[16,22]
**Nitrofurantoin**	-3.34	0.11	11	-3.29	0.06	2	[16,22]
**Norfloxacin**	-2.88	0.16	18	-2.75	0.15	1	[22]
**Nortriptyline**	-3.94	0.23	3	-3.93	0.02	2	[16,22]
**Ofloxacin**	-1.37	0.14	6	-1.27	0.04	1	[16]
**Olanzapine**	-4.47	0.17	1	-4.23	0.18	1	[24]
**Orphenadrine**	-3.71	0.35	1	-3.17	0.15	1	[22]
**Paliperidone**	-4.56	0.18	1	-4.31	0.22	1	[28]
**Papaverine**	-4.40	0.14	8	-4.21	0.24	4	[16,21,22,24]
**Phenazopyridine**	-3.99	0.16	6	-4.19	0.07	1	[16]
**Phenol,4-Iodo-**	-1.83	0.07	1	-1.72	0.04	1	[22]
**Phenylbutazone**	-4.51	0.36	10	-4.39	0.04	1	[22]
**Phenytoin**	-4.08	0.13	29	-3.86	0.18	1	[16]
**Phthalic_Acid,2-**	-1.46	0.02	10	-1.55	0.08	2	[16,22]
**Pindolol**	-3.81	0.14	6	-3.64	0.13	3	[22,24,28]
**Pioglitazone**	-6.21	0.81	3	-6.16	0.46	1	[28]
**Piroxicam**	-4.46	0.26	16	-4.73	0.06	4	[16,21,22]
**Pramoxine**	-3.46	0.39	1	-3.02	0.15	1	[22]
**Probenecid**	-4.82	0.24	3	-4.86	0.04	1	[17]
**Procaine**	-2.59	0.46	2	-1.72	0.07	1	[22]
**Prochlorperazine**	-4.38	0.26	1	-4.75	0.15	1	[22]
**Promethazine**	-4.39	0.19	10	-4.26	0.15	1	[22]
**Propranolol**	-3.82	0.30	4	-3.49	0.06	6	[21-24,27,28]
**Pyrimethamine**	-3.96	0.57	3	-4.11	0.22	1	[22]
**Quinine**	-3.16	0.67	5	-2.80	0.01	2	[16,22]
**Ranitidine**	-1.70	0.57	1	-2.50	0.26	1	[16]
**Rosiglitazone**	-5.28	0.50	3	-5.22	0.61	1	[28]
**Salicylic_Acid**	-1.88	0.08	20	-1.93	0.04	1	[17]
**Sertraline**	-5.41	0.28	2	-4.83	0.15	1	[22]
**Sulfacetamide**	-1.50	0.05	5	-1.52	0.04	1	[16]
**Sulfamerazine**	-3.10	0.07	6	-3.12	0.05	1	[22]
**Sulfamethazine**	-2.63	0.27	6	-2.73	0.04	1	[16]
**Sulfamethizole**	-2.77	0.14	5	-2.78	0.14	1	[17]
**Sulfasalazine**	-6.47	0.08	7	-6.21	0.10	2	[16,22]
**Sulfathiazole**	-2.61	0.23	8	-2.69	0.15	1	[22]
**Terfenadine**	-7.68	0.71	10	-8.40	0.15	1	[29]
**Tetracaine**	-3.22	0.11	1	-3.05	0.06	2	[16,24]
**Tetracycline**	-3.29	0.05	6	-3.00	0.09	2	[16,29]
**Tetracycline,Oxy-**	-3.27	0.07	4	-3.09	0.33	1	[16]
**Thiabendazole**	-4.13	0.46	3	-3.48	0.10	1	[17]
**Thymol**	-2.18	0.05	3	-2.19	0.04	1	[22]
**Tolbutamide**	-3.55	0.11	5	-3.50	0.05	2	[17,28]
**Tolmetin**	-4.05	0.15	1	-4.11	0.03	2	[16,22]
**Trazodone**	-3.31	0.09	4	-3.19	0.40	2	[17]
**Trichlormethiazide**	-3.64	0.12	3	-3.38	0.18	3	[16,22]
**Trimethoprim**	-2.64	0.39	9	-2.95	0.15	1	[16]
**Verapamil**	-4.36	0.28	9	-4.07	0.22	3	[21,22]
**Warfarin**	-4.77	0.22	9	-4.80	0.14	2	[22,24]

^a^ *SD* refers to the standard deviation of reported values from *N* different sourcs. The average *inter*laboratory *SD_i_* values for for the two sets of data are: *SD*_i_^SSF^ = 0.24 and *SD*_i_^CS^ = 0.15 log unit.
